# Tibial tubercle transfer SurgeRy and physiothErapy Versus physIotherapy only for chronic paTellofemorAL paIn: study protocol for a randomiSed controllEd trial (REVITALISE)

**DOI:** 10.1186/s12891-024-08226-y

**Published:** 2025-01-15

**Authors:** Myrthe P. F. van de Ven, Martin Ophey, Victor van de Graaf, Sebastiaan A. W. van de Groes, Marijn Sinkeldam, Charlotte H. W. Wijers, Sander Koëter

**Affiliations:** 1 Department of Orthopaedics, Canisius Wilhelmina Ziekenhuis, Nijmegen, The Netherlands; 2 Department of Orthopaedic Surgery and Sports Medicine, Amsterdam UMC, Amsterdam, The Netherlands; 3 Department of Orthopaedic Surgery, Bergman Clinics, Braillelaan 10, Rijswijk, 2289 CM The Netherlands; 4 Department of Orthopaedics, Radboud University Medical Centre, Nijmegen, The Netherlands; 5 CWZ Academy, Canisius Wilhelmina Ziekenhuis, Nijmegen, The Netherlands

**Keywords:** Patellofemoral pain, Tibial tubercle transfer, Physiotherapy, Randomised controlled trial

## Abstract

**Background:**

Patellofemoral pain (PFP) is a common musculoskeletal disorder resulting in anterior knee pain. Physiotherapy is the current standard treatment, while surgical intervention (tibial tubercle transfer [TTT]) is reserved for chronic cases when nonoperative treatment has failed. TTT can result in clinically meaningful improvement in patients with patellofemoral maltracking without instability. However, to date, no randomised controlled trials (RCT) have compared TTT combined with a physiotherapy (PT) programme to PT alone as the initial treatment for PFP.

**Methods:**

A single-centre nonblinded RCT in 40 patients with chronic PFP (> 1 year) and tibial tubercle-trochlear groove (TT-TG) > 15 mm will be randomised to either TTT followed by PT or to PT alone in a 1:1 ratio in a concealed manner. The primary outcome is knee pain at 18 weeks for the TTT group (6 weeks splint phase + 12 weeks PT) and at 12 weeks for the PT group, measured by the visual analog scale (VAS). Secondary outcomes include Patient-Reported Outcome Measures (PROMs) using the Kujala score, International Knee Documentation Committee (IKDC) ‘Subjective Knee Form’, Tegner Activity score and EuroQol 5D-5 L for quality of life. Range of motion (ROM) is measured using the decline step-down test (DSDT). Between-group differences will be analysed using multivariable linear regression analysis, while longitudinal differences will be assessed with linear mixed models for repeated measures. The difference in direct medical costs will also be assessed.

**Discussion:**

The REVITALISE study is the first randomised study to compare surgical intervention (TTT) followed by PT to PT alone in patients with chronic PFP. This study will generate level-1 evidence on the most effective treatment for chronic PFP, which can be integrated into the national guideline to aid orthopaedic surgeons and physiotherapists in their decision-making and ultimately improve our care for patients with chronic PFP.

**Trial registration:**

Study Identifier NCT06227806, registered on 26 Jan 2024 on ClinicalTrials.gov. The study underwent independent peer review and received approval from the ethical review board (number NL80956.091.22).

**Supplementary Information:**

The online version contains supplementary material available at 10.1186/s12891-024-08226-y.

## Introduction

### Background and rationale

Patellofemoral pain (PFP) is one of the most common forms of anterior knee pain, with a reported prevalence of 15–45% in physically active adolescents and adults, and it is more commonly diagnosed in women [1–3]. The symptoms are aggravated by activities involving loading the patellofemoral joint during weight-bearing on a flexed knee [4]. Additionally, psychological factors are influencing the experience of pain and are also related to physical activity [5]. A relatively large proportion of patients (40%) diagnosed with PFP continue to experience symptoms one to six years after diagnosis [6–9].

The treatment goals for PFP are to reduce pain, improve patellofemoral tracking and alignment, and return to the pre-injury level of activities. The conservative treatment of PFP with physiotherapy for the quadriceps and hip muscles as the cornerstone has shown varying degrees of success, from no difference [10, 11] to improvement [8, 12, 13]. To date, surgical intervention is not recommended in the international PFP consensus statement because high-level evidence about its effects is lacking [8]. In the Dutch guideline on ‘anterior knee pain’, a multidisciplinary stepped care treatment strategy model is incorporated, consisting of education and an exercise programme [14]. However, if symptoms persist after 12 weeks, the effect of further exercise therapy or surgery is unknown.

Carlson et al. [15] reported that the tibial tubercle-trochlear groove (TT-TG) distance was significantly greater in patients with PFP. In patients with patellar dislocation, a TT-TG > 15 mm is considered a clinically relevant anatomical substrate that negatively affects patellofemoral tracking and stability [15, 16]. According to a recent meta-analysis, tibial tubercle transfer (TTT) can lead to good results with clinically meaningful improvement in patients with patellofemoral maltracking without instability [17], while arthroscopy did not [18, 19]. It is considered a relatively safe procedure with a low complication rate [20, 21]. However, no randomised controlled trials (RCT) analysing the value of TTT or comparing TTT with physiotherapy for the treatment of chronic PFP have been published.

To further clarify the additional value of surgery in PFP, a RCT will be conducted to compare TTT in conjunction with a physiotherapy (PT) programme versus a PT alone in patients with chronic PFP and a TT-TG > 15 mm.

### Objectives

The primary objective of this study is to compare the between-group difference in knee pain over time for TTT followed by PT versus PT alone in patients aged 18–35 years with chronic PFP. We hypothesise that TTT combined with PT will result in a larger decrease in knee pain than PT alone.

Secondary objectives are to compare the between-group and longitudinal difference in change in (1) knee function, measured by validated Patient-Reported Outcome Measures (PROMs), (2) range of motion (ROM), (3) quality of life (QoL), (4) the functional limitations, and (5) the direct medical costs.

The results of this study can be used to develop and implement a better stepped-care treatment strategy for PFP and can be integrated into the national guideline for “anterior knee pain” [14].

## METHODS

### Study design and setting

A single-centre RCT. All patients will be recruited and included at the Orthopaedics Department of the Canisius Wilhelmina Hospital (CWZ), Nijmegen, the Netherlands. Eligible patients are randomised into two equal groups:

- The TTT group: 20 PFP patients who will be treated with TTT at the hospital of randomisation, followed by a 12-week supervised PT protocol;
- The PT group: 20 PFP patients who will be treated with the same 12-week supervised PT protocol.

This study will be conducted according to the principles of the Declaration of Helsinki [22] and in accordance with the Medical Research Involving Human Subjects Act (WMO), Good Clinical Practice (GCP) and the General Data Protection Regulation. The study will also be carried out in accordance with local legal requirements. This study was approved by the independent Ethical Committee METC Oost-Nederland (protocol number NL80956.091.22) and was registered on ClinicalTrials.gov (study identifier NCT06227806). Written informed consent will be provided by each participant before the start of the study assessments or the intervention. This protocol adheres to the SPIRIT checklist items.

### Participants

Participants between 18 and 35 years of age with at least 12 months of PFP and a tibial tubercle trochlear groove (TT-TG) distance ≥ 15 mm on CT or MRI will be recruited at the CWZ hospital in Nijmegen, The Netherlands. PFP is defined as:

- Pain around or behind the patella aggravated by at least one activity that loads the patellofemoral joint (e.g., squatting, running, or jumping);
- Pain during daily life activities, such as: standing for an extended period, walking, or walking upstairs or downstairs.

Participants will be excluded when one or more of the following exclusion criteria are met:

- Previous knee surgery;
- Reported ligamentous or meniscal injuries of the knee;
- A history of patellar dislocation (i.e. patellar instability), based on a positive apprehension test by physical examination and/or signs of instability on MRI, such as injury or rupture of the medial patellofemoral ligament [23]. However, subjects with patellar subluxation are included in the study;
- Malrotational deformity of the lower extremity evident from physical examination;
- Disabling general illness;
- Other diagnosed knee injuries, either clinically (such as jumper’s knee) or radiologically (such as osteochondritis dissecans);
- Patients who cannot undergo surgery;
- Pregnancy;
- Patients with inability to complete follow-up or with limited understanding of the Dutch language.

### Patient recruitment

All eligible patients will be approached by their treating physician at the orthopaedic department of the CWZ, provided with adequate patient information, and handed out a patient information file (PIF). They will be instructed about the TTT, PT, and possibility to crossover after 12 weeks of the PT. After having had sufficient time (> 24 h) to reconsider participation, they will be contacted by a researcher to obtain approval and plan a baseline visit where participants will sign informed consent. Patients will also be asked for permission to approach them for future related research. A list of all eligible patients will be recorded in a screening log, including those that do not wish to participate, as well as the reason if provided.

### Randomisation

Patients will be enrolled in the study by the study coordinator in the orthopaedic department. All patients who consent to participate in the study and meet the inclusion criteria will be randomised. Randomisation will be performed by the study coordinator after consent is signed and baseline data is collected. Patients will be randomised to the TTT + PT: PT only in a 1:1 ratio. The randomisation process will be carried out using a computer-generated randomisation method in the Castor electronic data capture (EDC) system [24], in random blocks of 4. The exact randomisation algorithm is unknown to any of the investigators; hence, concealment of allocation is successful in all patients.

### Blinding

Neither the treatment providers nor the study coordinator will be blinded due to the study design (with a blatant intervention).

### Interventions

#### Tibial tubercle transfer (TTT)

The TTT is a surgical procedure that realigns the patella and, as a result, improves patellar tracking in the trochlear groove. TTT is a relatively safe procedure with a low complication rate (< 1% for all major complications) [20, 21]. The TTT will be scheduled as soon as possible after randomisation, with a target of < 2 weeks. All surgeries will be carried out by either SK or Dr. S. Tigchelaar, both of whom have extensive experience in treating patellofemoral knee disorders. The operative technique used is described previously [25]. Patients will be discharged the same day after surgery. Then, a splint phase is started for 4–6 weeks. In this period, patients are instructed to use crutches for weight bearing while wearing the splint. ROM will be slowly built up under supervision of a physiotherapist. The physiotherapist will be instructed to contact the study coordinator when the PT begins (see below). After 6 weeks, an outpatient visit with the orthopaedic surgeon is scheduled for a check-up following regular care. At 6 months, there will be an outpatient visit following regular care for evaluation of possible symptoms caused by the hardware. Consequently, removal of hardware (ROH) can be performed if the patient experiences pain derived from the hardware and if complete bone healing is observed on radiographs.

#### Physiotherapy (PT) programme

This will start as soon as possible in the PT group and after the splint phase in the TTT group; the PT group will not wear a splint. The PT will consist of three levels and will be supervised by a physiotherapist. According to predefined criteria, the physiotherapist decides whether a patient can step onto the next level within the PT. The PT consists of flexibility, neuromuscular coordination, and strength exercises for the quadriceps and hip muscles. The PT is developed by a team of experienced physiotherapists who are specialised in treating patients with PFP and based upon the most recent physiotherapeutic insights [8]. In addition to training, education about PFP is incorporated into the PT according to the most recent insights regarding pain mechanisms [26, 27]. Physiotherapists who participate as self-chosen treating health care professionals by patients will be requested to follow an instructional video developed by the REVITALISE study team. Additionally, the three levels of exercise of the PT programme are available on the Physitrack^®^ tool for both participating physiotherapists and patients with PFP to improve exercise adherence and support clinical decision-making. In total, there will be 12 sessions with the physiotherapist. In the first three weeks of the PT, patients visit the physiotherapist twice a week. This will occur once a week from week 3 to week 6. This will occur every other week from week 6 to week 12. However, the treating physiotherapist may increase or decrease the number of appointments if needed. Additionally, the physiotherapist may adjust the exercises, sets, repetitions, weight and duration of loading tailoring to the specific needs of the patient. Since it is our intention that the PT is adjusted to the needs of each individual patient, the exact adherence and deviations to the protocol will not be monitored specifically. However, if necessary, we will consult with the treating physiotherapist in case the patient is not compliant. Moreover, the physiotherapist will be contacted by the study coordinator at the end of the PT to inquire about possible feedback on the PT and patient adherence and progression. The PT is included in the supplementary material (Supplementary file 1), which contains more detailed information and instructions.

#### TTT and PT versus PT only

The study involves four outpatient visits (at inclusion/randomisation, 6 weeks after the start of the intervention, after 12 (PT group)/18 weeks (surgery + PT group), and after 26 weeks in both groups). A physical examination will take place at every outpatient visit, with the addition of the decline step-down test (DSDT) at baseline and 12/18 weeks after randomisation. Patients will complete online questionnaires at every outpatient visit and 12 months after randomisation.

After randomisation, patients will either have a TTT (treatment group) or start with the PT (control group). From 12 weeks, there will be the possibility to crossover from the PT group to surgery if the symptoms do not improve. The criteria for this are a persisting high level of pain and patient-reported lack of effect of the PT. In that case, surgery will be performed within 2 weeks. Participants will then follow the same protocol as the TTT group, including the PT programme, surveys, and physical measurements.

#### Criteria for discontinuing or modifying allocated interventions

Subjects can leave the study at any time for any reason if they wish to do so without any consequences. The investigator can decide to withdraw a subject from the study for urgent medical reasons. There will be no follow-up of patients who withdraw from the study, but reasons for withdrawal will be recorded. Patients who withdraw from the study will not be replaced.

#### Strategies to improve adherence to interventions

To improve adherence, patients will receive the PT in text and with visual instructions on the Physitrack app if possible. They will also receive a weekly questionnaire to register the amount of exercising with the physiotherapist and at home, as well as their VAS score during the 12-week PT.

#### Relevant concomitant care permitted or prohibited during the trial

Patients are permitted to take any medication such as paracetamol or other prescribed painkillers. The use of other interventions, such as braces or insoles, is not restricted.

#### Provisions for post-trial care

Post-trial care will follow standard care, especially for the surgery group. Therefore, we do not expect post-trial harm from participation in this study. There are no specific provisions for post-trial care.

### Outcomes

#### Primary outcome

The main study parameter will be the between-group difference in activity-related knee pain at 12 weeks after PT in both groups, measured with the 10 cm VAS-scale. The point in time is different for the two groups, since there is a 6 week splint phase incorporated in the TTT group. Thus, knee pain will assessed at week 18 in the TTT group (6 weeks for the TTT and splint + 12 weeks of PT) and at week 12 in PT group. To compare both treatments as reliably as possible, we opted to not set this outcome measure further in time, due to the possibility for cross-over from the PT group to the TTT group. Furthermore, we expect that a plateau phase of pain will be reached within this period. The mean from the two VAS pain scores in week 12 (PT group) and 18 (TTT group) by the weekly questionnaire and outpatient visit will be used to account for variance in pain levels. Participants will assess the maximum pain they had felt during the previous 7 days when participating in activities of daily living such as ascending stairs, descending stairs and standing up from a sitting position. The 10-cm VAS is a valid and responsive tool for evaluating overall pain in patients with PFP [28]. A minimal clinically important difference (MCID) of 2 cm or 20% has been reported to reflect real changes in a patient’s symptoms [28].

#### Secondary outcomes

Secondary outcomes are between-group difference in: pain during the intervention (VAS); functional outcomes (Kujala score, IKDC), activity level (Tegner score), quality of life (EQ-5D-5 L), and functional ROM (DSDT) at 12/18 weeks follow-up, as well as the change over time from baseline to 12 months follow-up. Furthermore, this study will provide a global overview of the direct medical costs of surgery + PT compared to PT only treatment.

### VAS

In the TTT group, it could be possible that the trajectory of recovery might affect VAS pain scores at week 18 still. Therefore, a secondary outcome is the between-group difference in VAS pain score over time, which enables us to assess whether the VAS score at 12/18 weeks is stable or changes over time. This will be from baseline to 12 months follow-up, also including the VAS pain score in the weekly questionnaire during the PT.

### Functional outcomes

The Kujala score is a 13-item questionnaire used to evaluate subjective symptoms and functional limitations in patients with PFP [29]. Kujala grade ranges from 0 to 100, with 100 reflecting no signs of PFP. This index shows high validity and is responsive to change [28]. To detect improvement, a change of 8–10 points is required [28].

The International Knee Documentation Committee (IKDC) ‘Subjective Knee Form’ is a tool containing 11 items: 7 on knee symptoms, 2 on function, and 2 on sports activities, resulting in a score ranging from 0 to 100, with 100 reflecting the highest level of pain and functional restrictions. The IKDC has been validated in people with PFP [30]. The IKDC showed high internal consistency (0.92) and test-retest reliability (0.95), with a value change of 9 points indicating true change [31].

### Activity level

The Tegner activity score consists of 1 item, which scores the level of work- and sport activities on a 10-point scale, with 10 reflecting a high level of activity [32].

### Quality of life

The health-related quality of life (EQ-5D-5 L) questionnaire consists of 5 dimensions (mobility, self-care, usual activities, pain/discomfort, and anxiety/depression) that are scored on 5 levels (no problems, slight problems, moderate problems, severe problems, and extreme problems) [33]. An index value will be calculated to reflect health status on a scale of 0 to 1, where 1 represents maximum quality of life.

### Functional ROM

The maximum pain-free flexion angle (MPFFA) is measured by the DSDT, which was performed according to the instructions mentioned elsewhere [34]. The DSDT is a reliable and valid performance test simulating stair descent with almost perfect intra- and interobserver reliability (*ICC* 0.83–0.85) and an average positive correlation between the DSDT score and the total AKPS score (*r*_*s*_ = 0.31, *p* = .030) [34].

#### Other study parameters

This study will include items strongly recommended by the International Patellofemoral Research Network (iPFRN) in the REPORT-PFP consensus statement [35]. These include demographics, such as age, biological sex, symptom duration, and unilateral/bilateral symptoms [35]. Furthermore, physical examination data, such as height and weight (and hence BMI) will be recorded. To evaluate the adherence to the PT, patients will also receive a weekly email during the PT asking how often they have visited the physiotherapist and how often they have executed the PT at home.

Any adverse events (AEs) will be monitored, as explained in the ‘Adverse event reporting and harms’ section. The rate of ROH after surgery will be registered as a separate outcome.

### Participant timeline

**Fig. 1:**
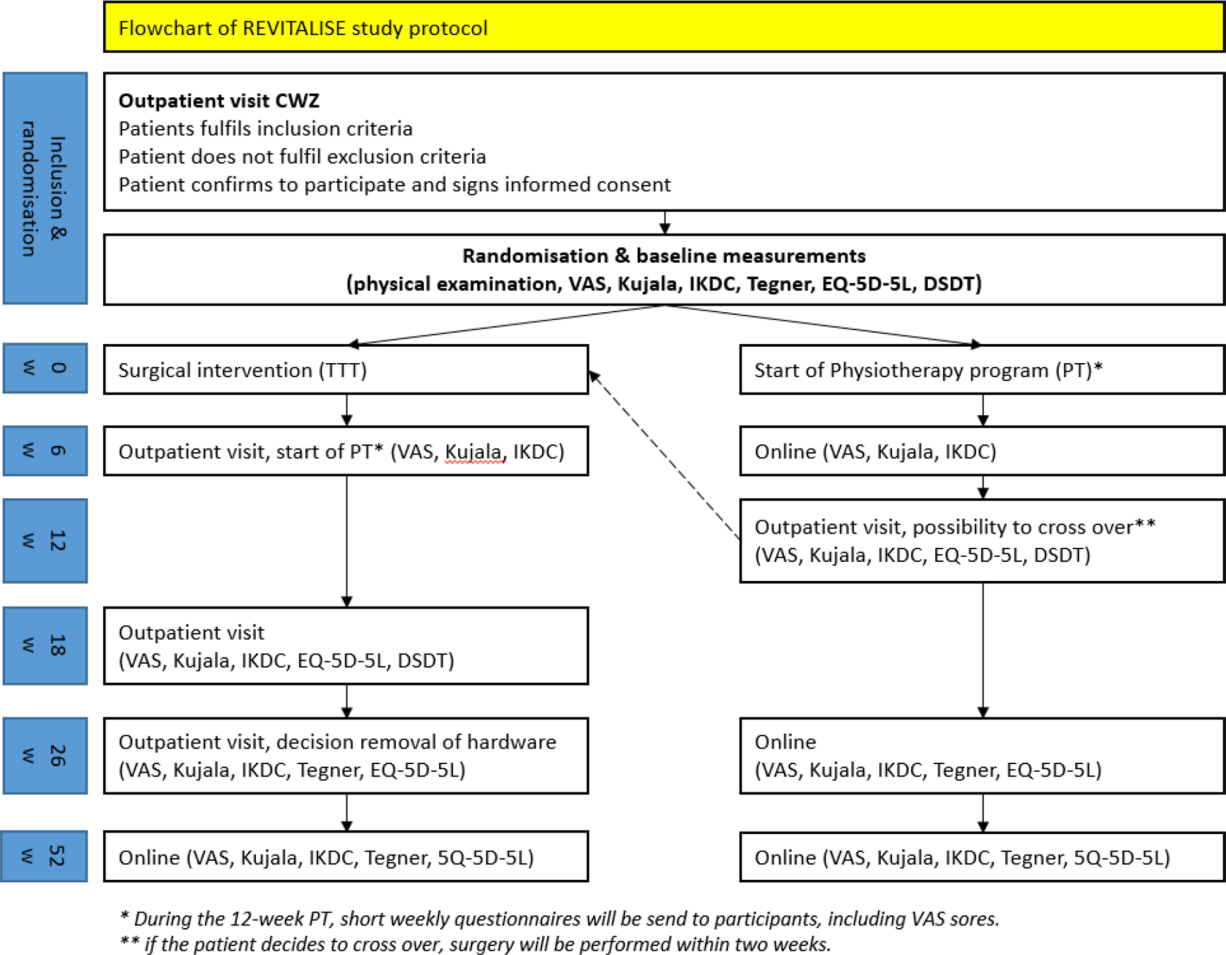
Flowchart of the study protocol and procedures

Figure 1 provides an overview of the study design and procedures.

### Sample size

Our primary outcome is the between-group difference in overall knee pain 12 weeks after PT and 18 weeks after TTT and PT, measured with the VAS score. Our hypothesis is that the improvement in the TTT group will be greater than the improvement in the PT group.

To detect the superiority of the treat-to-target TTT with PT to PT alone, we calculated the required sample size for an independent samples t-test on the following assumptions: power (1-beta) 0.8, significance level (alpha) 0.05, and two-sided testing. Effect size was set at the MCID for VAS-pain, based on a study by Crossley et al., who demonstrated that a change in the VAS score of two points or more is considered clinically relevant and worthwhile for performing surgery [28]. Based on a study by Bayoumi et al., which compared different surgical techniques for patellar maltracking without instability, the anticipated mean for the PT only group was 6.2 (reflecting the pre-operative value), and SD was 2.0 [17]. Based on these parameters, a sample size of 16 patients per group is needed. Anticipating on a loss to follow-up of 20%, 20 patients per group are needed.

### Feasibility of recruitment

Given the > 50 TTTs for patients with chronic PFP already performed annually in the CWZ, and accounting for patients who are not willing to participate, we expect to have sufficient numbers of patients for our inclusion within 24 months. The CWZ is a nationally recognized top clinical expertise centre in (patellofemoral) knee surgery and performs approximately 80% of patellofemoral surgeries in the Netherlands, including TTT [36].

### Data collection methods

Plans for assessment and collection of outcomes.

The clinical assessment data and PROMs data will be collected in CastorEDC. This data collection will be organized by the study coordinator.

Plans to promote participant retention and complete follow-up.

Participants will be closely monitored by the study coordinator to ensure follow-up. To reduce patient burden, data collection will be web-based as often as possible.

### Data management

We have created a data management plan in line with the General Data Protection Regulation, which can be viewed upon request. The data will be managed within the online CastorEDC system. This is a secure cloud-based platform that contains an audit trail, automatic range checks, and pseudomised study IDs for all data.

### Confidentiality

The type of data that is collected is in line with the General Data Protection Regulation. Study IDs are pseudonymised in CastorEDC for all data. The key to the study ID is safely kept by the study coordinator. The data will be stored for 15 years.

### Statistical methods for primary and secondary outcomes

In accordance with the iPFRN, the REPORT-PFP consensus statement outcome measure description will be adequate [35]. The items described in the assessment will be in adequate detail to allow replication. We will report *n* (%), the mean (SD), and median (interquartile range; IQR) for categorical/descriptive, normally distributed continuous and non-normally distributed continuous data, respectively [35]. Results over time will be visualized descriptively in spaghetti plots. The primary analyses will be performed following the intention-to-treat method.

Statistical analyses will be done using *R* software (R Foundation for Statistical Computing, Vienna, Austria).

#### Primary endpoint

The primary outcome parameter is the between-group difference in VAS score, at 12 weeks after start of the PT (week 18 for TTT group). For analysis of the primary parameter, the mean of the two VAS scores at 12 weeks of the PT in the TTT group will be compared with that of the PT group. Baseline VAS score, biological sex, and treatment group will be included as covariates in a multivariable linear regression analysis.

#### Secondary endpoints

For the secondary parameters (functional outcomes on the Kujala and IKDC score, activity level on Tegner score, QoL on the EuroQol-5D-5 L score, and functional ROM on the DSDT score) at 12 weeks after the start of the PT (week 18 for TTT group), the between-group difference will be assessed using multivariable linear regression analysis in the same manner as the primary endpoint.

To assess the longitudinal clinical effectiveness of both groups, linear mixed models for repeated measures (MMRM) will be used for the secondary outcomes and activity-related knee pain on VAS score during the PT from baseline to 12 months follow-up. MMRMs take the correlation of multiple measurements over time within one patient into account. In the MMRM, these secondary outcomes will be analysed as dependent variables. To evaluate the effect at different time points, the fixed effects will be patient, time, and the treatment group (TTT versus PT only). Time will be included as dummy variable, with baseline as reference.

The main effects for treatment will be reported as difference in means with 95% confidence intervals (CI).

#### Other study parameters

The economic aspect will be published based upon an estimation of the direct medical healthcare costs for TTT + PT and conservative care for PFP + PT, including costs for hospital visits, scans, surgery, physiotherapy, prescription medication, and possible readmission. The medical costs are based on resource costs. Furthermore, ROH and complications will be reported.

#### Methods for additional analyses (e.g., subgroup analyses)

Per-protocol analyses will be performed including only the patients that did not crossover from the PT to TTT group, for comparison with intention-to treat analysis.

Methods in analysis to handle protocol non-adherence and any statistical methods to handle missing data.

Patient adherence to the PT will be extracted from the weekly questionnaire and will be reported. To test whether this affects the primary outcome, adherence will be added as extra covariate in the multivariable linear regression analysis as sub analysis.

In the case of missing outcome values, data will be imputed before analysis using multiple imputation methods.

Plans to provide access to the full protocol, participant-level data and statistical code.

The trial protocol provided to the METC is available from the corresponding author upon reasonable request. Data will be handled according to the FAIR principles (a guideline to improve the Findability, Accessibility, Interoperability, and Reusability of Digital Assets) [37]. After completion of the study, metadata will be available upon request. For access to anonymised participant-level data and statistical code, an application can be submitted to the corresponding author.

### Monitoring

#### Data monitoring

A data monitoring plan has been created and is available upon request. As both groups in this study are within the spectrum of regular care, no data safety monitoring board is installed. Furthermore, since this is a monocentre study, a coordinating centre is not needed. The CWZ Academy acts as a Trial Steering Committee in this study to provide independent oversight and yearly checks.

No interim analyses will be performed. As the treatments in both groups are within the range of usual care, we do not anticipate differences between the groups that could warrant early cessation of the study due to detrimental effects to the participant.

#### Adverse event reporting and harms

All AEs reported spontaneously by the subject, observed by the investigator, or his staff will be recorded. The patient’s general practitioner will receive a letter about the patient’s participation in the study. To prevent under registration of AEs, we will ask for this information during outpatient visits. AEs will be reported until 12 weeks for the PT group and 18 weeks for the surgery group.

Elective hospital admission will not be considered a serious adverse event (SAE). Prolongation of initial hospital stay after surgery due to pain or reduced mobility and the need for mobilisation under sedation (hardly ever performed after TTT) will not be considered a serious adverse event. The investigator will report all SAEs to the sponsor without any delay after obtaining knowledge of the events. The sponsor will report the SAEs through the web portal ToetsingOnline to the accredited METC, which approved the protocol within 7 days of first knowledge for SAEs that result in death or are life threatening, followed by a period of a maximum of 8 days to complete the initial preliminary report. All other SAEs will be reported within a maximum of 15 days after the sponsor has first knowledge of the serious adverse events. SAEs will be reported until 12 weeks for the PT group and 18 weeks for the surgery group.

#### Auditing

Monitoring will be performed once per year. It will involve monitoring the investigator site file, informed consent, SAEs, in- and exclusion criteria, among others. Monitoring will be performed according to Nederlandse Federatie van Universitair Medische Centra (NFU) guidelines and a monitoring plan by a monitor from the CWZ academy.

#### Protocol amendments

All amendments will be reported to the METC that gave a favourable opinion. Non-substantial amendments will not be notified to the accredited METC or the competent authority but will be recorded and filed by the investigator.

#### Dissemination plans

Overall trial results will be communicated to participants, presented at (inter)national conferences, and reported in peer-reviewed journals. There are no publication restrictions. Medically relevant incidental findings will be shared with the study subject.

## Discussion

The REVITALISE study is the first RCT to compare surgical intervention followed by PT to PT alone in patients with chronic PFP. This study’s results will gain insight into the most effective treatment for patients with chronic PFP and will enhance our treatment of this high burden disease with currently a high variability of provided care [14]. This variation seems to be not or weakly associated with patient characteristics, elucidating the need for a more standardised approach to prevent over- or under treatment. The study will answer the question whether patients receiving TTT followed by PT have less pain and better functional outcomes than those receiving PT alone. In addition to measuring the effectiveness of TTT plus PT versus PT alone, a range of other outcomes will be assessed (e.g., physical function, complications, direct medical costs), to provide a broader view of the effect of both treatments.

The strengths of the study include the randomised controlled design supported with multiple involved professions. Physiotherapists are involved in the design of the PT. Orthopaedic surgeons are involved in the TTT protocol. Since this is a single-centre study, little or no variation in surgical technique is expected.

This study has several limitations. First, the crossover design potentially affects long-term (i.e., > 12 weeks) comparisons of the groups, as well as induces bias in cases where patients become less motivated to follow physiotherapy. However, as the primary analysis is according to the intention-to-treat principles, we do not expect this to have a major impact. Second, at the time of presentation at the hospitals, patients will have had different treatments, i.e., the care given by the family physician, the outpatient physiotherapist, or other involved professionals. In our physiotherapy programme, special care is given to education of the patient about PFP, as well as a focus on building balance and strength in multiple muscle groups, not only the quadriceps. Third, for logistical reasons, it is not possible for all participants to receive physiotherapy from the same physiotherapist. To limit protocol variations, we will contact each physiotherapist to explain the research design, the PT, and the need to adhere to this programme. Simultaneously, the physiotherapist should tailor the programme to the participants, as their individual needs and limitations are always the highest priority.

The results of this study will address an important evidence gap and will have a significant impact on the care of patients with chronic PFP.

### Trial status

The study is currently open for inclusion. Recruitment started on 24 October 2023, and the expected end date of recruitment is 24 October 2025.

## Electronic supplementary material

Below is the link to the electronic supplementary material.

Supplementary Material 1

Supplementary Material 2

## Data Availability

Data will be handled according to FAIR principles. After completion of the study, metadata will be available upon request. For access to participant-level data and statistical code, an application can be submitted to the corresponding author. The PT used in the current study is available as supplementary material (see Supplementary file 1).

## Abbreviations

AE: Adverse event
AKPS: Anterior Knee Pain Score (Kujala score)
BMI: Body Mass Index
CI: Confidence Interval
CT: Computer Tomography (scan)
CWZ: Canisius Wilhelmina Ziekenhuis
DSDT: Decline step down test
EDC: Electronic data capture
EQ-5D-5L: Questionnaire for measuring health-related quality of life
GCP: Good Clinical Practise
IKDC: International Knee Documentation Committee Subjective Knee Form
iPFRN: International Patellofemoral Research Network
IQR: Interquartile range
MCID: Minimal clinically important difference
METC: Medical research ethics committee
in Dutch: Medisch-ethische toetsingscommissie
MMRM: Mixed model repeated measures
MPFFA: Maximum Pain-Free Flexion Angle
MRI: Magnetic Resonance Imaging (scan)
NFU: Nederlandse Federatie van Universitair Medische Centra
PFP: Patellofemoral Pain
PROMS: Patient Reported Outcome Measures
PT: Physiotherapy programme
QoL: Quality of Life
RCT: Randomised controlled trial
ROH: Removal of Hardware
ROM: Range of movement
SAE: Serious adverse event
SD: Standard deviation
TTT: Tibial Tubercle Transfer
TT-TG: Tibial tubercle trochlear groove
VAS: Visual analog scale
WMO: Medical Research Involving Human Subjects Act
In Dutch: Wet Medisch-wetenschappelijk Onderzoek
