# Supplementary material for: Tibial tubercle transfer SurgeRy and physiothErapy Versus physIotherapy only for chronic paTellofemorAL paIn: study protocol for a randomiSed controllEd trial (REVITALISE)
Source: BMC Musculoskelet Disord. 2025 Jan 15;26:53. doi: 10.1186/s12891-024-08226-y (PMC11734609; doi:10.1186/s12891-024-08226-y)
Supplement: Supplementary file 1 — Supplementary Material 1 [file 12891_2024_8226_MOESM1_ESM.pdf]

Bijlage B: toestemmingsformulier deelname REVITALISE

- Ik heb de informatiebrief gelezen. Ook kon ik vragen stellen. Mijn vragen zijn goed genoeg beantwoord. Ik had genoeg tijd om te beslissen of ik meedoe.
- Ik weet dat meedoen vrijwillig is. Ook weet ik dat ik op ieder moment kan beslissen om toch niet mee te doen met het onderzoek. Of om ermee te stoppen. Ik hoef dan niet te zeggen waarom ik wil stoppen.
- Ik weet dat voor de controle van het onderzoek sommige mensen al mijn gegevens kunnen inzien. Ik geef deze mensen toestemming om mijn gegevens in te zien voor deze controle.
- Ik weet dat de onderzoeker mijn huisarts zal informeren over deelname aan het onderzoek.
- Ik geef toestemming om mijn gegevens 15 jaar na afloop van dit onderzoek te bewaren. Zodat dit misschien gebruikt kan worden voor een nieuw onderzoek.

**Door onderstaande vragen te beantwoorden met ja of nee, geef ik wel of geen toestemming:**		
Ik geef de onderzoeker toestemming om mijn huisarts of specialist informatie te geven over onverwachte bevindingen uit het onderzoek die van belang zijn voor mijn gezondheid.	Ja ☐	Nee ☐
Ik geef de onderzoekers toestemming om mijn gegevens te verzamelen en gebruiken.	Ja ☐	Nee ☐
Ik geef toestemming om mij eventueel na dit onderzoek te vragen of ik wil meedoen met een vervolgonderzoek.	Ja ☐	Nee ☐
Ik wil meedoen aan dit onderzoek.	Ja ☐	Nee ☐

Mijn naam is (proefpersoon): ....

Handtekening: .... Datum : __ / __ / __

- Ik verklaar dat ik deze proefpersoon volledig heb geïnformeerd over het genoemde onderzoek.
- Als er tijdens het onderzoek informatie bekend wordt die de toestemming van de proefpersoon zou kunnen beïnvloeden, dan breng ik hem/haar daarvan tijdig op de hoogte.

Naam onderzoeker (of diens vertegenwoordiger):.....................................

Handtekening:........................... Datum: __ / __ / __

De proefpersoon krijgt een volledige informatiebrief mee, samen met een getekende versie van het toestemmingsformulier.
