# Supplementary material for: Tibial tubercle transfer SurgeRy and physiothErapy Versus physIotherapy only for chronic paTellofemorAL paIn: study protocol for a randomiSed controllEd trial (REVITALISE)
Source: BMC Musculoskelet Disord. 2025 Jan 15;26:53. doi: 10.1186/s12891-024-08226-y (PMC11734609; doi:10.1186/s12891-024-08226-y)
Supplement: Supplementary file 2 — Supplementary Material 2 [file 12891_2024_8226_MOESM2_ESM.pdf]

Physiotherapy Program REVITALISE

'Surgery and exercise versus exercise only for chronic patellofemoral pain syndrome: a randomised controlled trial.'

CWZ Department of Orthopaedics | orthopedie@cwz.nl |cwz.nl/expertisecentrumknie

CWZ has top clinical expertise in the field of complex knee complaints, in particular patello femoral surgery.

Program

The physiotherapist, in consultation with the patient, determines the number of exercises, sets, repetitions, weight, and duration of loading. The rehabilitation program includes target numbers, which are suggestions.

Key criteria are (1):

- The number of repetitions and sets of an exercise can be performed 'qualitatively well' by the patient. This is assessed by the physiotherapist. 'Qualitatively well' means that the patient can maintain the instructed range of motion, movement rhythm, and movement speed during the specified number of repetitions and sets. It also means that the patient does not perform compensatory movements such as 'kneeing-in' or loss of tension in the starting or ending position.
- The patient can complete the specified number of repetitions and sets.
- The patient does not experience an increase in knee pain after or the next morning. For operated patients, it is also essential that there is no increase in hydrops or pain at the level of the tibial tuberosity during training, after, or the next morning.

Note: These criteria are mandatory and rigorously monitored by the physiotherapist.

Number of treatments

- For the first three weeks, there will be two treatments per week.
- The following three weeks will involve one treatment per week.
- In the last six weeks, there will be one treatment every two weeks.
- In total, there will be twelve treatments. The treating physiotherapist may adjust this number if necessary based on their judgment.

Phases

The program consists of 5 phases. After phase 0, the splint phase, the patient initially enters phase 1, the 'Mobility and Education Phase.' If these exercises are feasible and meet the criteria below, exercises from phase 2, the 'Training Build-up Phase,' can be gradually integrated into the program. Depending on the specific clinical presentation, the physiotherapist may choose to focus more on education, stretching and massage, or conscious quadriceps control. The physiotherapist aims for progression but takes the specific clinical presentation and the criteria below as a starting point. If exercises from phase 2 are well-executed and meet the criteria below, exercises from phase 3, the 'Functional Training Phase,' can be gradually integrated into the program.

Purpose and background of the program

The goal of this program is to provide a structured guideline for the rehabilitation of patients with chronic PFP or patients with a history of tibial tuberosity transfer (TTT). This means that the patient does not necessarily have to go through all phases in the 12 weeks. Ensure that patients with a painful knee do not overload it. At the same time, patients responding well to the program can be progressively loaded over the 12 weeks.

Additionally, the Education component in phase 1 is essential. It is important for the patient to understand that there is no structural damage, but the front of the knee is very sensitive (sensitization). The longer this process has been ongoing, the larger the painful area often becomes. This can also explain why no abnormalities are detected on the MRI (except a possible enlarged TT-TG distance). Due to knee pain, the patient may move differently, or perhaps move less. This has an impact on mobility, coordination, and strength throughout the body. This physiotherapy program aims to reverse this process, without ignoring the knee's excessive sensitivity. ADL (Activities of Daily Living) and sports activities must also be adjusted to avoid post-pain.

Physiotherapy Program

This program is built on experiences and guidelines in the CWZ (Orthopaedics Department) and the most recent Dutch guideline concerning patellofemoral pain).

Phase 0

Splint phase
(TTT group)
1-4 weeks

Functional level

- Passive mobilizations patellofemoral.
- Passive mobilizations flexion guided by pain. The goal is 90° after 4 weeks.
- Motor reactivation of the quadriceps muscle (possibly with electrostimulation), starting with isometric exercises, progressing to guided active exercises.
- Active exercises, gluteal and calf musculature.

Activity and participation level

- Splint Policy: Weeks 1-4, weight-bearing mobilization (standing and walking) is only allowed with the splint. In rest, if necessary, for pain relief. Weeks 5-6, gradual reduction of splint use, but only if pain symptoms at the level of the tibial tuberosity do not increase.
- Neuromuscular training with qualitatively correct execution.
- Optimizing the walking pattern, both when wearing the splint and during the reduction of splint use. Finding the right balance in the progression of load and crutch use.

Aberrant course

- If the wound does not close or an infection occurs.
- If there is a significantly reduced flexion of <30° after 6 weeks.
- If progressing to full load is not successful. Attention should be given to an increase in pain symptoms at the level of the tibial tuberosity.
- If there is no voluntary control of the quadriceps muscle after 4-6 weeks.

Criteria transition to phase 1

- Good wound healing.
- No knee pain.
- Minimal hydrops/synovitis.
- Normal patellar mobility (left = right).
- Extension 0° and flexion at least 90°.
- Voluntary contraction of the quadriceps muscle.
- Qualitatively correct execution of neuromuscular exercises in phase 1.

A check with the orthopedic surgeon takes place after 4 weeks.
It is not necessary to wait for this check before starting phase 1.

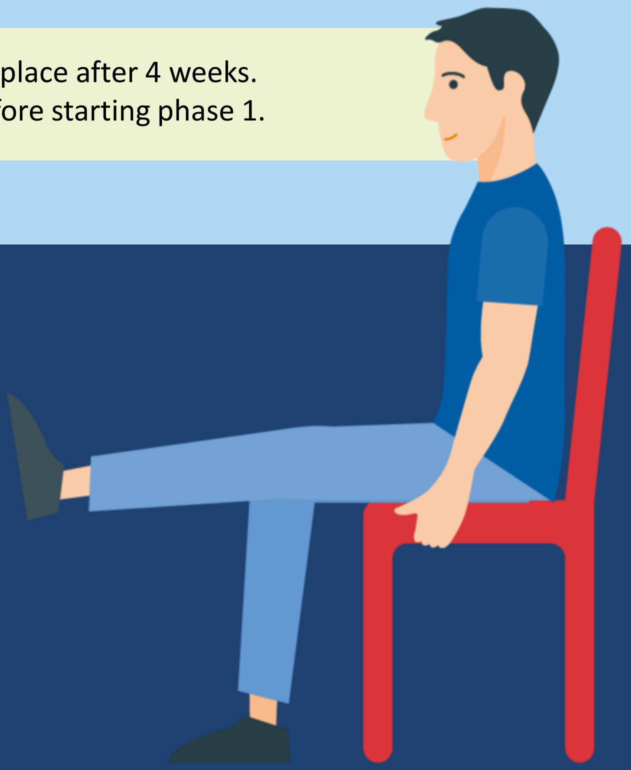

The physiotherapist will contact the researcher to discuss the criteria for transitioning to phase 1 and to indicate when phase 1 will be initiated.

Phase 1

Mobility and education

Week 1-4

If transition criteria are met

Education

- 'Where is the pain coming from.'
  Explanation about the role of a 'good night's rest' and 'loading/load-bearing capacity'.
  - Ducth guideline anterior knee pain exercise therapy and additional therapy
  - Example information regarding PFP in adolescents
  - Example flyer education regarding PFP (Figure 1 in article)

Activities / exercises

Activities / exercises	Working towards
• Stretching of quadriceps/iliopsoas, hamstrings, and iliotibial tract.	**3x 60 sec**
• Self-massage as preferred; this can also be supported by hands-on mobilizations/massage from the physiotherapist.	**3-5 min**
• Conscious quadriceps control (Sitting with legs extended on the ground or a bench, consciously contracting and relaxing the quadriceps, paying attention to compensations such as the lower back or hip).	**3-5 min**
• Bilateral calf raises.	**3 x 20 reps**
• Side-lying clam hip exercise.	**3 x 20 reps**
• Aerobic training (cycling with low resistance, can also be arm cycling).	**1 x 10 min**

Patients from the operated group can additionally receive 'hands-on' treatment to improve arthrogenic joint mobility (patellofemoral or tibiofemoral). In addition, if there are limitations in extension/flexion, additional exercises such as heel slides and prone hangs can be implemented.

Phase 2

Training (endurance)

Week 5-8

Activities / exercises

Activities / exercises	Working towards
• Conscious dynamic knee control in the early and late stance phase.<br>◦ Early stance phase: co-contraction of quadriceps/hamstring, can be expanded in the next phase to a pushback lunge.<br>◦ Late stance phase: consciously relax in the final extension.	**3-5 min**
• Straight leg raise, alternating left and right.	**10 x 20 sec**
• Calf raise on a step.	**3 x 20 reps**
• Glute bridge, bilateral.	**10 x 20 sec**
• Wall sit, bilateral at 30 or 45 degrees.	**10 x 20 sec**
• Pelvic drop exercise.	**3 x 20 reps**
• Plank.	**3 x 30 sec**
• Side plank, both sides.	**3 x 15 sec**
• Further expand aerobic training.	

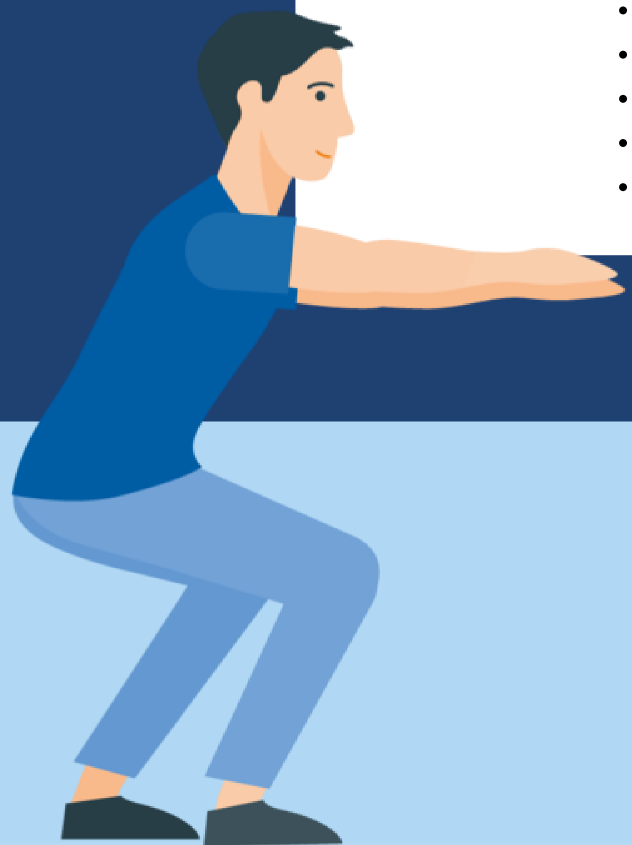

Phase 3

Functional training
Week 9-12

Activities / exercises

Activities / exercises	Working towards
• Build conscious eccentric knee control from light to full range of motion pushback lunge (focus on quality!).	**3 x 20 reps**
• Concentric/eccentric calf raises on a step.	**3 x 20 reps**
• Wall sit, bilateral at 90 degrees.	**10 x 20 sec**
• Bodyweight squat.	**3 x 15-20 reps**
• Bodyweight step-up.	**3 x 15-20 reps**
• Bodyweight split squat, both feet on toes.	**3 x 15-20 reps**
• Single-leg bridge with the other leg extended.	**3 x 20 reps**
• Aerobic training (cycling, if possible around aerobic threshold).	**2 x 10 min**
• Possibly add upper extremity strength exercises such as lat pull-down or chest press.	**3 x 10 reps**

The aim in this phase is to increase the intensity of the traditional quadriceps-oriented exercises in the strength rehabilitation method "intensive strength endurance." It is also possible in this phase to intensify the more hip-oriented exercises from phase 2 to the point of training "intensive strength endurance" (2). Avoid overloading patients who are not ready for these steps in this phase but ensure that patients capable of handling this are optimally and progressively loaded.

Criteria transition to phase 4

- Qualitatively correct execution of neuromuscular exercises in phase 3.
- LSI (Limb Symmetry Index) > 80% for strength of quadriceps and hamstrings.
- LSI > 80% for hop test battery.
- Administering the IKDC and/or KOOS assessments.

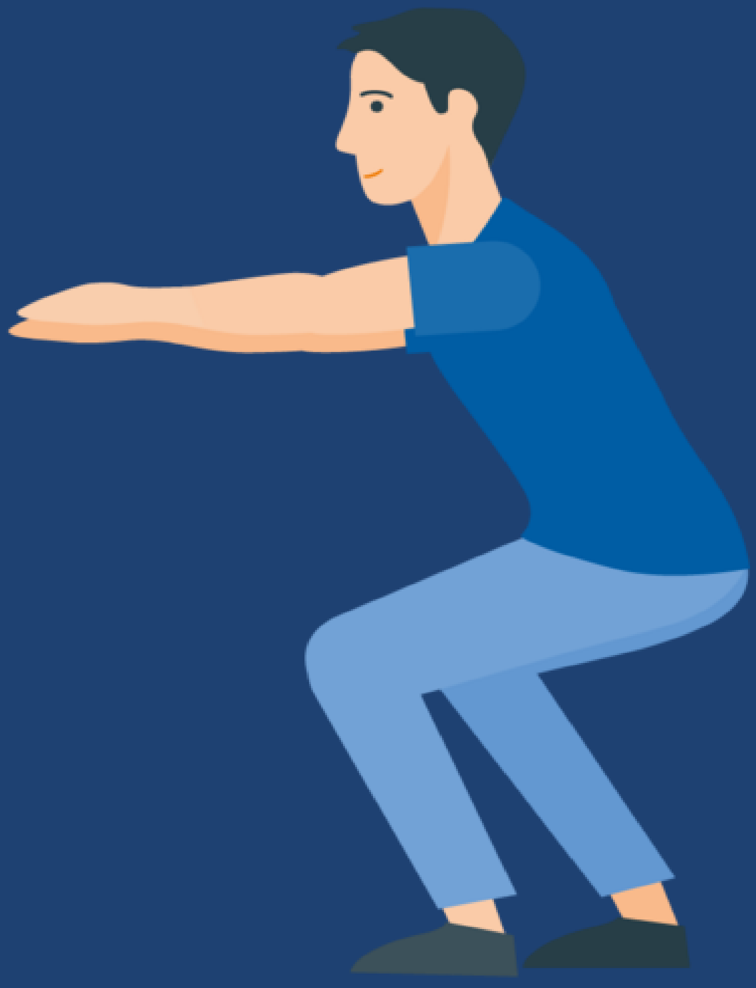

Phase 4

Sport specific

> 12 weeks

If transition criteria are met (could also be earlier than 12 weeks)

Functional level

- Maintaining full ROM
- Intensifying (sport) specific muscle strengthening exercises.

Activity and participation level

- Expanding neuromuscular training with qualitatively correct execution.
- Expanding strength training in full ROM.
- Expanding jogging/cycling to sports-specific loads.
- Expanding and intensifying agility training.
- Resuming training at the personal sports club, if knee capacity allows.

Criteria terminating revalidation

- Correct walking pattern, symmetric running pattern, and qualitatively correct execution of sports-specific movements.
- LSI > 90% for strength of quadriceps and hamstrings.
- LSI > 90% for hop test battery, supplemented with the single-leg hop and hold test.
- Drop jump with observation/video analysis of movement quality.
- Administering the IKDC and/or KOOS assessments.

Frequency of physiotherapy program

The exercises should be performed at least twice a week. If there is no post-exercise pain, this can be expanded to every other day. Motivated, pain-free patients may continue repeating the training from the previous phase. There are no two training sessions per day, and there is at least one rest day per week.

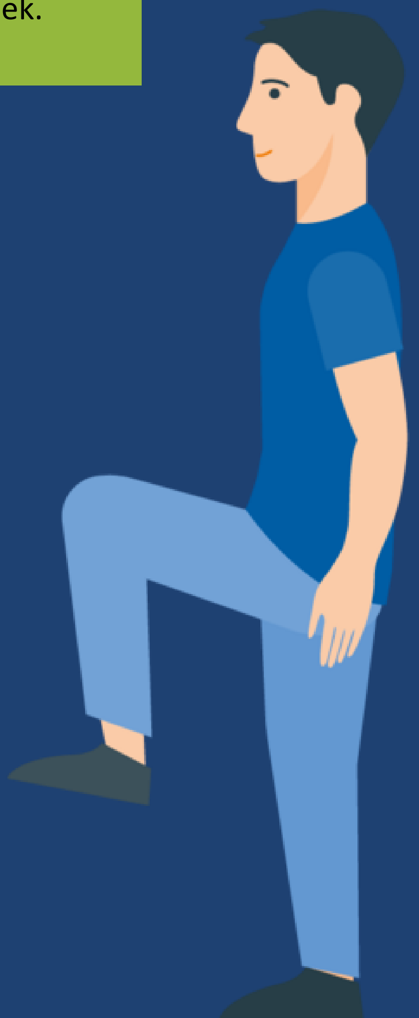

References

(1) Rathleff MS, Roos EM, Olesen JL, Rasmussen S. Early intervention for adolescents with patellofemoral pain syndrome--a pragmatic cluster randomised controlled trial. BMC Musculoskelet Disord. 2012 Jan 27;13:9. doi: 10.1186/1471-2474-13-9. PMID: 22280484; PMCID: PMC3328242.

(2) Hansen, R., Brushøj, C., Rathleff, M. S., Magnusson, S. P., & Henriksen, M. (2023). Quadriceps or hip exercises for patellofemoral pain? A randomised controlled equivalence trial. British Journal of Sports Medicine, bjsports-2022-106197. https://doi.org/10.1136/BJSPORTS-2022-106197
